# Impact of a Nationwide Lockdown on SARS-CoV-2 Transmissibility, Italy

**DOI:** 10.3201/eid2701.202114

**Published:** 2021-01

**Authors:** Giorgio Guzzetta, Flavia Riccardo, Valentina Marziano, Piero Poletti, Filippo Trentini, Antonino Bella, Xanthi Andrianou, Martina Del Manso, Massimo Fabiani, Stefania Bellino, Stefano Boros, Alberto Mateo Urdiales, Maria Fenicia Vescio, Andrea Piccioli, COVID-19 Working Group,, Silvio Brusaferro, Giovanni Rezza, Patrizio Pezzotti, Marco Ajelli, Stefano Merler

**Affiliations:** Fondazione Bruno Kessler, Trento, Italy (G. Guzzetta, V. Marziano, P. Poletti, F. Trentini, S. Merler);; Istituto Superiore di Sanità, Rome, Italy (F. Riccardo, A. Bella, X. Andrianou, M. Del Manso, M. Fabiani, S. Bellino, S. Boros, A. Mateo Urdiales, M.F. Vescio, A. Piccioli, S. Brusaferro, G. Rezza, P. Pezzotti);; Cyprus University of Technology, Limassol, Cyprus (X. Andrianou);; European Centre for Disease Prevention and Control, Stockholm, Sweden (M. Del Manso, A. Mateo Urdiales);; Indiana University School of Public Health, Bloomington, Indiana, USA (M. Ajelli);; Northeastern University, Boston, Massachusetts, USA (M. Ajelli)

**Keywords:** coronavirus disease, SARS-CoV-2, severe acute respiratory syndrome coronavirus 2, severe acute respiratory syndrome, SARS, viruses, respiratory infections, zoonoses, COVID-19, lockdown, reproduction number, transmissibility, Italy

## Abstract

On March 11, 2020, Italy imposed a national lockdown to curtail the spread of severe acute respiratory syndrome coronavirus 2. We estimate that, 14 days after lockdown, the net reproduction number had dropped below 1 and remained stable at »0.76 (95% CI 0.67–0.85) in all regions for >3 of the following weeks.

On February 21, 2020, the earliest known case of locally transmitted severe acute respiratory syndrome coronavirus 2 (SARS-COV-2) infection was reported in Italy (*1*; D. Cereda et al., unpub. data, https://arxiv.org/abs/2003.09320). Since then, several interventions have been deployed to control disease spread in regions with sustained transmission, including quarantine of most-affected municipalities, ban of mass gatherings, and local school closures. School closure at the national level was mandated on March 5, and a national lockdown (stay-home mandate and closure of all nonessential productive activities) was issued on March 11 (*2**,**3*), then eased after May 4, 2020 (Appendix). The aim of this study is to evaluate the impact of these interventions on SARS-CoV-2 transmissibility in Italy.

## The Study

We measured SARS-CoV-2 transmissibility in terms of the basic (R_0_) and net (R_t_) reproduction numbers. These quantities represent the mean number of secondary infections generated by 1 primary infector in a fully susceptible population (R_0_) and in the presence of control interventions and human behavioral adaptations (R_t_). When R_t_ decreases below the threshold of 1, the number of new infections begins to decline. Estimates were obtained through a Bayesian approach applied to case-based surveillance data collected by regional health authorities (Appendix).

To account for the geographic heterogeneity in contacts, healthcare organization, and timelines of interventions, R_t_ was estimated separately for different provinces and regions. We considered all 19 regions in Italy plus the 2 autonomous provinces of Trento and Bolzano. Moreover, we considered 100 of the remaining 105 provinces for which the data were sufficiently complete. The selected provinces covered 99.1% of the population of Italy and, as of May 3, 2020, accounted for 153,558 symptomatic cases (97.9% of the total recorded in the surveillance database). To evaluate the progressive decrease of transmission, we computed R_t_ at 3 dates: the day before lockdown (March 10) and 1 and 2 weeks after lockdown (March 18 and 25). In addition, we considered the average value of R_t_ over the successive 3 weeks (March 26–April 15). These choices were suggested by the trend of the national R_t_ (Appendix).

**Figure 1:**
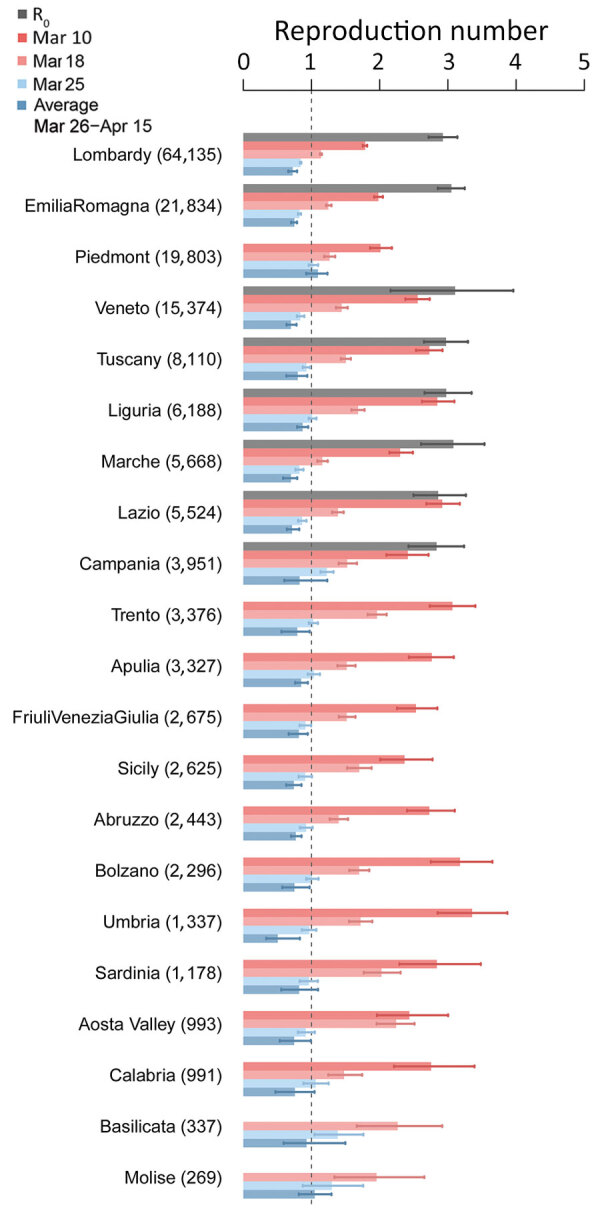
Basic (R_0_) and net reproduction numbers for severe acute respiratory syndrome coronavirus 2 for all regions and autonomous provinces in Italy. Regions are sorted by decreasing number of cases (numbers in parentheses) on April 17. Bars indicate mean numbers; error bars indicate 95% CIs.

The R_0_ range was 2.83–3.10 (Figure 1) in the 8 regions for which the estimate was possible (Appendix). On March 10, R_t_ range was 1.79–3.36 across regions; Basilicata and Molise had an insufficient number of symptomatic cases (Figure 1). One week into lockdown, on March 18, R_t_ had decreased consistently, but no region or autonomous province was yet below the epidemic threshold (Figure 1). As of March 25, R_t_ was <1 in most regions and autonomous provinces (12/21) and <1 in the successive 3 weeks for all regions except Molise and Piedmont (Figure 1). The mean value of R_t_ across the regions and autonomous provinces, weighted by the number of reported cases at the corresponding date, fell from an average of 2.03 (95% CI 1.94–2.13) on March 10 to 1.28 (95% CI 1.23–1.33) on March 18, to 0.88 (95% CI 0.84–0.91) on March 25, corresponding to an overall 62.6% reduction (range across regions 45.6%–85.0%). In the 3 weeks of March 26–April 15, R_t_ remained stable in all regions, showing a further slight reduction at an average value of 0.76 (95% CI 0.67–0.85).

**Figure 2:**
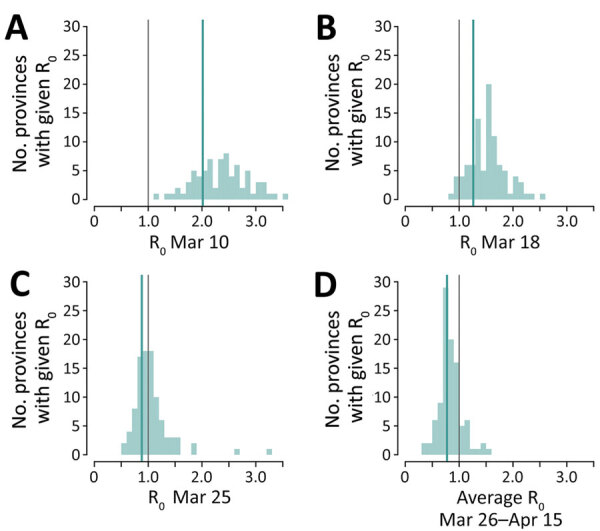
Distribution of the mean net reproduction numbers for severe acute respiratory syndrome coronavirus 2 in 100 selected provinces in Italy. Green lines indicate average value of R_t_, weighted by the number of reported cases by each province. Gray line indicates epidemic threshold.

Results were consistent when analyzing estimates from the 100 selected provinces (Figure 2). As of March 10, no province had a mean estimated value of R_t_ <1 (n = 75; the number of symptomatic cases was insufficient for the estimate in 25 provinces). One week after lockdown, on March 18, 5/93 provinces (5.4%) had an average R_t_ <1, whereas on March 25 this figure increased to 49/96 provinces (51.0%). The fraction of provinces with R_t_ below 1 rose to 84/100 (84.0%) when considering the average over the following 3 weeks. The mean value of the reproduction number across the provinces, weighted by the province’s number of reported cases at the corresponding date, was 2.01 (95% CI 1.83–2.22) on March 10, 1.26 (95%CI 1.15–1.38) on March 18, 0.88 (95% CI 0.79–0.97) on March 25, and 0.77 (95% CI 0.63–0.95) for the period March 26–April 15.

## Conclusions

Our results suggest that the national lockdown put in place as of March 11 to limit the spread of SARS-CoV-2 in Italy brought R_t_ below 1 in most regions and provinces within 2 weeks. Although R_t_ had been declining steeply even before the national lockdown (*3*) in regions with intense interventions, we estimated that the epidemic was brought under control only after the implementation of the lockdown. Lockdown was fundamental to prevent an explosion in the number of cases in other regions in which transmission had started weeks later compared with the outbreak epicenter (Lombardy, Veneto, Emilia Romagna). The range of estimates of R_0_ in 8 regions was 2.8–3.1, within the range of estimates obtained for other countries (*4**–**6*).

A massive and sustained scale-up of testing capacity was set up in all regions of Italy during the course of the epidemic (*7*); it was not accompanied by a corresponding increase of confirmed incident cases in the weeks following March 25, as indicated by the declining proportion of positive tests (Appendix). This finding suggests an increase of notification rates and thus a possible overestimation of R_t_ (*8*). To compensate for possible biases, we supplemented our results by computing alternative estimates based on the time series of hospitalized cases. Criteria for hospitalization are more homogeneous across local health systems and over time than testing criteria because they are grounded in the patient’s need for medical assistance. Furthermore, the hospitalization date is an easier piece of information to collect with respect to the symptom onset date, which requires an epidemiologic investigation and may be subject to recall bias. Results obtained with this additional method were consistent with our conclusions (Appendix).

We did not consider asymptomatic cases in our analysis. The adopted methodology is robust even in the presence of large underdetection rates, provided that these rates are constant over time or even slightly fluctuating (*8*,*9*). We did not consider imported cases either, due to the lack of data; imported cases are potential infectors, but do not contribute to the number of transmitted cases, thereby lowering estimates of reproduction numbers. In Italy, most cases were probably locally transmitted. After March 11, the ban of movement across provinces imposed by the lockdown made the role of imported cases negligible. Reproduction numbers were computed using the distribution of serial interval for Italy (*10*; D. Cereda et al.), which is an acceptable approximation of the generation interval (*11*; S. Hu et al., unpub. data, https://10.1101/2020.07.23.20160317). Both distributions are strongly influenced by country-dependent variables, such as behavior of infected persons and the adopted interventions. Estimates of the generation interval distribution are still unavailable for Italy as of October 2020.

Italy was the first country outside of Asia to impose a nationwide lockdown, rapidly followed by many countries worldwide. The effectiveness of lockdown had been proven in China, where the reproduction number was estimated to fall to »0.3 in Wuhan (*12*) and 0.5 in other provinces (*8*); Western countries had enforced a comparatively softer version of restrictions. We have shown that these measures enabled rapid reversal of the epidemic trend within 2 weeks, although probably at higher values of the reproduction number.

Appendix Additional information about the impact of a nationwide lockdown on SARS-COV-2 Transmissibility, Italy.
