# Supplementary material for: Impact of a Nationwide Lockdown on SARS-CoV-2 Transmissibility, Italy
Source: Emerg Infect Dis. 2021 Jan;27(1):267–70. doi: 10.3201/eid2701.202114 (PMC7774526; doi:10.3201/eid2701.202114)
Supplement: Appendix — Additional information about the impact of a nationwide lockdown on SARS-COV-2 Transmissibility, Italy. [file 20-2114-Techapp-s1.pdf]

Article DOI: https://doi.org/10.3201/eid2701.202114

Impact of a Nationwide Lockdown on SARS-CoV-2 Transmissibility, Italy

Appendix

Timeline of Interventions

The interventions performed in Italy to control the spread of severe acute respiratory syndrome coronavirus 2 (SARS-CoV-2) were initially localized in the 3 regions in which the large majority of cases had been detected (Lombardy, Veneto, Emilia Romagna), and included the creation of red zones in areas with sustained transmission, the ban of mass gatherings, and the closure of schools. School closure at the national level was mandated on March 5. On March 8, the red zone was extended to the entire region of Lombardy, and to several provinces in the regions of Emilia-Romagna, Piedmont, Veneto, and Marche. Finally, the national lockdown (stay-home mandate and closure of all nonessential productive activities) was issued on March 11, 2020. The timeline of interventions performed over the period February 23, 2020−May 4, 2020 is summarized (Appendix Table 1).

Changes in Infection Ascertainment Rates

Temporal changes in the ascertainment rate of SARS-CoV-2 infections can be indirectly evaluated by observing changes in the proportion of positive tests, given by the ratio between the number of new confirmed cases and the number of performed tests (*1*). A declining proportion of positive tests may derive from the combined effect of a nondecreasing prevalence of infection and an increasing number of tests being administered, thereby resulting in higher rates of cases being ascertained. A massive scale-up of the testing capacity was implemented during the course of the epidemic, resulting in a declining proportion of positive tests after March 25; however, the decline of positive tests needs to be interpreted in the context of a likely declining incidence (R_t <1) (Appendix Figure 1).

Bayesian Methods for Estimating the Basic and Net Reproduction Number

Case-based surveillance data were collected by regional health authorities and collated by the Istituto Superiore di Sanità using a secure online platform, according to a progressively harmonized track record. Data include, among other information, the place of residence, the date of symptom onset and the date of first hospital admission for laboratory-confirmed COVID-19 cases (*3*). In the early phase of the epidemics, the Italian regions did not report the number of cases that were imported from abroad or from other regions in the country. However, it is likely that the large majority of cases were locally transmitted, given that the epidemic was already widespread by the time of detection on February 1, 2020. After March 11, the national lockdown imposed a ban on movement across provinces except for well-documented special cases (health- or work-related), and thus the role of imported cases was probably negligible.

The distribution of the net reproduction number R_t was estimated by applying a well-established statistical method (*4*), which is based on the knowledge of the distribution of the generation time and on the time series of cases. In particular, the posterior distribution of R_t for any time point t was estimated by applying the Metropolis-Hastings Markov chain Monte Carlo sampling to a likelihood function defined as follows:

$$\mathcal{L} = \prod_{t=1}^{T} P\left(C(t);\ R_t \sum_{s=1}^{T} \varphi(s) C(t-s) \right)$$

where

• $P(k; \lambda)$ is the probability mass function of a Poisson distribution (i.e., the probability of observing k events if these events occur with rate λ)

• $C(t)$ is the daily number of new cases having symptom onset at time t

• R_t is the net reproduction number at time t to be estimated

• $\varphi(s)$ is the probability distribution density of the generation time evaluated at time s.

As a proxy for the distribution of the generation time, we used the distribution of the serial interval, estimated from the analysis of contact tracing data in Lombardy (D. Cereda et al., unpub. data, https://arxiv.org/abs/2003.09320), i.e., a gamma function with shape 1.87 and rate 0.28, having a mean of 6.6 days. This estimate was later confirmed by independent study on a

village (Vo' Euganeo) in the region of Veneto (*5*) and is within the range estimated for other countries (*6*–*9*; S. Hu et al., unpub. data, https://10.1101/2020.07.23.20160317).

To estimate R_0, we estimated a constant daily reproduction number $R_t = R_0$ over a time window, defined as a period of exponential growth in the early phase of the outbreak preceding the implementation of interventions (Appendix Table 2).

Regions that were not considered for the estimation of R_0 did not have a clearly identifiable exponential growth window of $\geq$1 week before the implementation of any interventions and $\geq$5 symptomatic cases per day. In the early phase of the epidemic, the region of Piedmont was not able to track the date of symptom onset for a large number of cases, resulting in an epidemic curve that cannot be used to provide a reliable estimate of R_0.

Trends in Country-Level Net Reproduction Number

We estimated the net reproduction number R_t from the time series of cases occurring in the whole country by date of symptom onset (Appendix Figure 2). Darker gray lines indicate the dates of March 10, 18, and 25, at which we sampled the regional and provincial estimates in the main analysis. A declining trend was visible before the lockdown (March 11), but lockdown enhanced the negative slope of the decline and brought R_t below threshold. After March 25, the R_t for Italy oscillated slightly around a stable value.

Comparison of Results with Hospitalization-Derived Reproduction Number

We performed a sensitivity analysis in which we adopted the same methodology used to estimate R_t, but applied to the time series of hospitalized cases (by date of hospitalization) instead of date of symptom onset (Appendix Figure 3). In particular, we estimated the reproduction number at March 25, using 2 different datasets: the time series of COVID-19 cases by date of symptom onset C(t) (estimate denoted by R^{symp}), as shown in Figure 1; and the time series of hospitalized cases by date of hospital admission, H(t) (estimate denoted by R^{hosp}). Because case-patients are admitted to the hospital at delayed time D from their symptom onset, we computed R^{hosp} using the shifted time series of hospitalized cases, H(t+D). The median value of D was estimated at 7 days from surveillance data, using 32,893 cases for which both the date of symptom onset and the date of hospital admission were available. Overall, H(t) includes

60,439 hospitalized cases recorded in the surveillance dataset as of April 1. Estimates of R^{hosp} for Piedmont could not be computed because the hospitalization data was incomplete.

References

1. Omori R, Mizumoto K, Chowell G. Changes in testing rates could mask the novel coronavirus disease (COVID-19) growth rate. Int J Infect Dis. 2020;94:116–18.

2. Dipartimento di Protezione Civile. COVID-19 Italy—Situation monitoring. Github [cited 2020 Oct 6]. https://github.com/pcm-dpc/COVID-19

3. Riccardo F, Ajelli M, Andrianou X, Bella A, Del Manso M, Fabiani M et al. Epidemiological characteristics of COVID-19 cases in Italy and estimates of the reproductive numbers one month into the epidemic. Euro Surveill. In press 2020.

4. Cori A, Ferguson NM, Fraser C, Cauchemez S. A new framework and software to estimate time-varying reproduction numbers during epidemics. Am J Epidemiol. 2013;178:1505–12. https://doi.org/10.1093/aje/kwt133

5. Lavezzo E, Franchin E, Ciavarella C, Cuomo-Dannenburg G, Barzon L, Del Vecchio C, et al. Suppression of a SARS-CoV-2 outbreak in the Italian municipality of Vo'. Nature. 2020;584:425–9. https://doi.org/10.1038/s41586-020-2488-1

6. Nishiura H, Linton NM, Akhmetzhanov AR. Serial interval of novel coronavirus (COVID-19) infections. Int J Infect Dis. 2020;93:284–6. https://doi.org/10.1016/j.ijid.2020.02.060

7. Wu JT, Leung K, Bushman M, Kishore N, Niehus R, de Salazar PM, et al. Estimating clinical severity of COVID-19 from the transmission dynamics in Wuhan, China. Nature Medicine, 2020;26:1149–50.

8. Li Q, Guan X, Wu P, Wang X, Zhou L, Tong Y, et al. Early transmission dynamics in Wuhan, China, of novel coronavirus–infected pneumonia. N Engl J Med. 2020;382:1199–207. https://doi.org/10.1056/NEJMoa2001316

9. Zhang J, Litvinova M, Wang W, Wang Y, Deng X, Chen X, et al. Evolving epidemiology and transmission dynamics of coronavirus disease 2019 outside Hubei province, China: a descriptive and modelling study. Lancet Infect Dis. 2020;20:793–802. https://doi.org/10.1016/S1473-3099(20)30230-9

Appendix Table 1. Interventions performed in Italy to prevent transmission of severe acute respiratory syndrome coronavirus 2 during February 21−May 4, 2020

Date	Location	Interventions
February 23	Lombardy	Creation of red zones in 11 municipalities of Lombardy and around the municipality of Vo' Euganeo in Veneto; ban of mass gatherings; school closure
	Veneto	
March 2	Emilia-Romagna	Ban of mass gatherings; school closure
March 5	All Italian regions	School closure
March 8	Lombardy	Closure of all non-essential productive activities; stay-home mandate except for well-documented special cases (health or work-related)
	5 provinces in Emilia-Romagna	
	5 provinces in Piedmont<br>3 provinces in Veneto<br>1 province in Marche	
March 11	All regions of Italy	Closure of all nonessential productive activities; stay-home mandate except for well-documented special cases (health or work-related)

Appendix Table 2. Regions characterized by periods of exponential growth before the national lockdown issued in Italy on March 11, 2020

Region	From	To
Campania	February 27	March 5
Emilia-Romagna	February 20	February 27
Lazio	February 27	March 5
Liguria	February 27	March 5
Lombardy	February 13	February 20
Marche	February 20	February 27
Toscana	February 27	March 5
Veneto	February 15	February 22

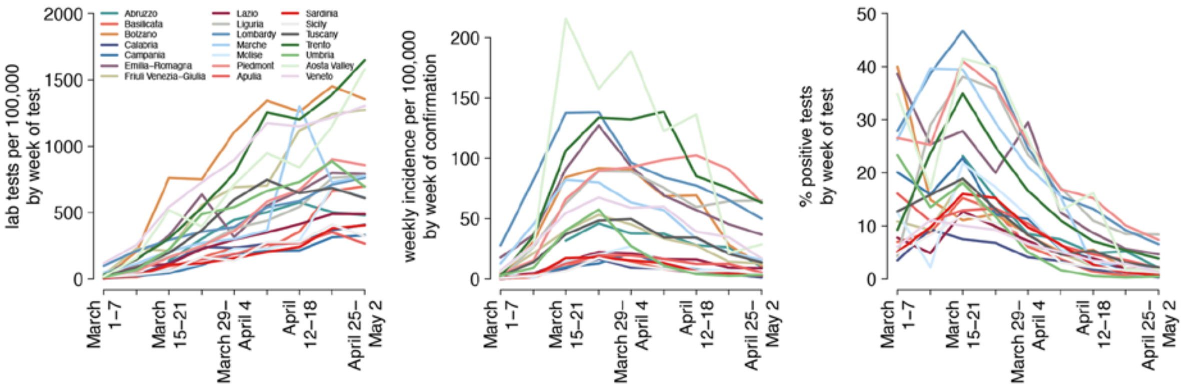

Appendix Figure 1. Number of lab tests (left) and lab-confirmed incident cases (center) per 100,000 population, and proportion of positive tests (right) in all regions and 2 autonomous provinces of Italy, as reported by the Italian Civil Protection Department (*2*). Note that lab-confirmed cases refer to infections occurring several days and up to few weeks before the reporting date, due to delays related to development of symptoms, seeking for medical care, execution of tests, and reporting to national authorities.

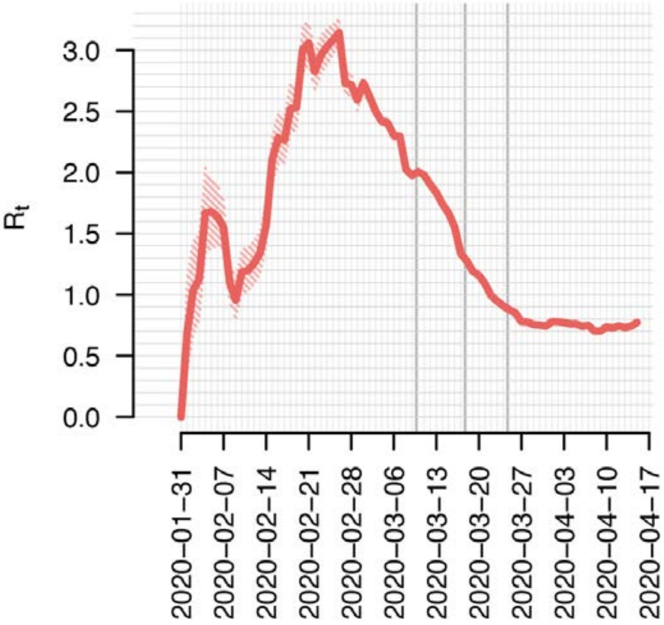

Appendix Figure 2. Estimates of the reproduction number over time, using the time series of COVID-19 cases in Italy by date of symptom onset.

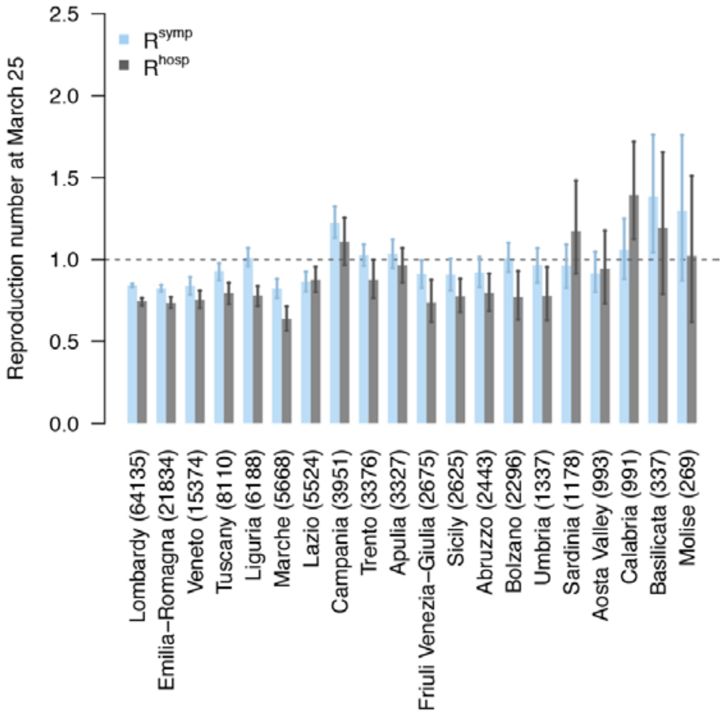

Appendix Figure 3. Estimates of the reproduction number at March 25, using the time series of COVID-19 cases by date of symptom onset (R^{symp}) and the time series of hospitalized cases by date of hospital admission (R^{hosp}).
